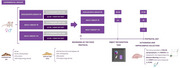# Effects of high‐fat diet on synaptic plasticity and astrocytic markers throughout development

**DOI:** 10.1002/alz70860_099788

**Published:** 2025-12-23

**Authors:** Letícia Cunha Pereira de Souza, Ariadni Mesquita Peres, Aline Cândida Ferreira, Ricardo Maia Dantas, Ana Caroline Silva Silveira, Giovana Barbosa Raphaelli, Mariana Cabral Lena, Eduardo da Silva Santos, Jéssica Hauschild Taday, Marina Concli Leite, Rachel Krolow Santos Silva Bast

**Affiliations:** ^1^ Universidade Federal do Rio Grande do Sul, Porto Alegre, Rio Grande do Sul, Brazil

## Abstract

**Background:**

The Central Nervous System (CNS) structure and functionality can be altered according to lived experiences. This plasticity is greater during childhood and adolescence, facilitating individual adaptation to the environment. However, it also increases CNS susceptibility to external factors, potentially compromising the developmental process.

**Method:**

On postnatal day (PND) 21, male and female Wistar rats were divided into three groups according to the developmental stage at which the outcomes would be evaluated: (1) adolescents 59, (2) adults 70, and (3) adults 110. Furthermore, the animals at each stage were allocated into two experimental groups according to the diet received: (1) control, received only a standard diet (SD), or (2) high‐fat diet (HFD), received both SD and HFD. Animals were kept on their respective diets throughout the experiment. Short‐term memory was assessed using an object recognition task. The immunocontent of synaptophysin and postsynaptic density protein (PSD‐95) was evaluated using the Western blotting technique. The levels of the glial fibrillary acidic protein (GFAP) were measured by ELISA.

**Result:**

Caloric consumption increased over time in the three stages evaluated (*p* <0.05), with weight differences between the control and HFD groups occurring only in adulthood (*p* <0.05). The discrimination index in object recognition diminished in males both in adolescence (*p* <0.05) and in early adulthood (*p* <0.05). In the hippocampus, for males, during adolescence, the immunocontent of PSD‐95 (*p* <0.05) and synaptophysin (*p* <0.05) increases. In early adulthood, DPN 70, the rise in synaptophysin is maintained (*p* <0.05). On PND 110 days, PSD‐95 and synaptophysin (*p* <0.05) increase. GFAP levels are high at both moments of the adult phase (*p* <0.05). For females, synaptophysin increases in adolescence (*p* <0.05). At 110 days, synaptophysin (*p* <0.05), PSD‐95 (*p* <0.05), and GFAP (*p* <0.05) are increased.

**Conclusion:**

Exposure to adverse external factors, such as HFD, during critical periods of development alters the astrocytic markers and synaptic plasticity, leading to reduced pruning, in both sexes, and these changes remain throughout life.